# Scaling Principles of White Matter Connectivity in the Human and Nonhuman Primate Brain

**DOI:** 10.1093/cercor/bhab384

**Published:** 2021-11-24

**Authors:** Dirk Jan Ardesch, Lianne H Scholtens, Siemon C de Lange, Lea Roumazeilles, Alexandre A Khrapitchev, Todd M Preuss, James K Rilling, Rogier B Mars, Martijn P van den Heuvel

**Affiliations:** Department of Complex Trait Genetics, Center for Neurogenomics and Cognitive Research, Vrije Universiteit Amsterdam, Amsterdam Neuroscience, 1081 HV, Amsterdam, the Netherlands; Department of Complex Trait Genetics, Center for Neurogenomics and Cognitive Research, Vrije Universiteit Amsterdam, Amsterdam Neuroscience, 1081 HV, Amsterdam, the Netherlands; Department of Complex Trait Genetics, Center for Neurogenomics and Cognitive Research, Vrije Universiteit Amsterdam, Amsterdam Neuroscience, 1081 HV, Amsterdam, the Netherlands; Department of Sleep and Cognition, Netherlands Institute for Neuroscience (NIN), an institute of the Royal Netherlands Academy of Arts and Sciences, 1105 BA Amsterdam, the Netherlands; Wellcome Centre for Integrative Neuroimaging, Department of Experimental Psychology, University of Oxford, Oxford OX1 3SR, UK; Medical Research Council Oxford Institute for Radiation Oncology, University of Oxford, Oxford OX3 7DQ, UK; Yerkes National Primate Research Center, Emory University, Atlanta, GA 30329, USA; Center for Translational Social Neuroscience, Emory University, Atlanta, GA 30329, USA; Department of Pathology and Laboratory Medicine, Emory University School of Medicine, Atlanta, GA 30307, USA; Yerkes National Primate Research Center, Emory University, Atlanta, GA 30329, USA; Center for Translational Social Neuroscience, Emory University, Atlanta, GA 30329, USA; Department of Anthropology, Emory University, Atlanta, GA 30322, USA; Silvio O. Conte Center for Oxytocin and Social Cognition, Emory University, Atlanta, GA 30322, USA; Department of Psychiatry and Behavioral Sciences, Emory University, Atlanta, GA 30322, USA; Donders Institute for Brain, Cognition and Behaviour, Radboud University Nijmegen, AJ 6525, Nijmegen, the Netherlands; Wellcome Centre for Integrative Neuroimaging, Centre for fMRI of the Brain (FMRIB), Nuffield Department of Clinical Neurosciences, John Radcliffe Hospital, University of Oxford, Oxford OX3 9DU, UK; Department of Complex Trait Genetics, Center for Neurogenomics and Cognitive Research, Vrije Universiteit Amsterdam, Amsterdam Neuroscience, 1081 HV, Amsterdam, the Netherlands; Department of Child Psychiatry, Amsterdam UMC, Vrije Universiteit Amsterdam, Amsterdam Neuroscience, 1081 HV, Amsterdam, the Netherlands

**Keywords:** allometry, connectome, evolution, neuroimaging, specialization

## Abstract

Brains come in many shapes and sizes. Nature has endowed big-brained primate species like humans with a proportionally large cerebral cortex. Comparative studies have suggested, however, that the total volume allocated to white matter connectivity—the brain’s infrastructure for long-range interregional communication—does not keep pace with the cortex. We investigated the consequences of this allometric scaling on brain connectivity and network organization. We collated structural and diffusion magnetic resonance imaging data across 14 primate species, describing a comprehensive 350-fold range in brain size across species. We show volumetric scaling relationships that indeed point toward a restriction of macroscale connectivity in bigger brains. We report cortical surface area to outpace white matter volume, with larger brains showing lower levels of overall connectedness particularly through sparser long-range connectivity. We show that these constraints on white matter connectivity are associated with longer communication paths, higher local network clustering, and higher levels of asymmetry in connectivity patterns between homologous areas across the left and right hemispheres. Our findings reveal conserved scaling relationships of major brain components and show consequences for macroscale brain circuitry, providing insights into the connectome architecture that could be expected in larger brains such as the human brain.

## Introduction

Brains show a great diversity in size. Brain volume in primates ranges from a few cubic centimeters in lemurs and galagos to around 1300–1400 cm^3^ in humans (Stephan et al. 1981; Schoenemann 2013). From smaller to larger brains, not all structures tend to keep similar proportions: Evidence from comparative postmortem and magnetic resonance imaging (MRI) studies suggests that brain structures can show disproportionate differences in size from smaller to larger brains, a phenomenon known as allometric scaling (Hofman 1989; Rilling and Insel 1999a). The cerebral cortex, for example, scales faster than expected based on total brain volume, resulting in a larger proportion of cortex in humans and other great apes compared with smaller primate species (Hofman 1989; Mota et al. 2019).

The effect of allometric scaling on brain connectivity, a key factor in shaping brain function (Sporns et al. 2005), remains largely unknown. Comparative studies have noted that the white matter tends to take up more and more space in larger-sized brains compared with smaller brains (Deacon 1990; Rilling and Insel 1999a; Hofman 2001): The proportion of white matter to total brain volume ranges from an estimated 11% in mice to 27% in macaques to 40–41% in chimpanzees and humans (Zhang and Sejnowski 2000). While the proportion of white matter is higher in larger brains, the cortical surface has been noted to scale even faster (Hofman 1989; Mota et al. 2019). An increasing number of cortical neurons has been theorized to quickly outpace the total space needed for axonal connections (Ringo 1991; Herculano-Houzel et al. 2007), suggesting a net decrease in corticocortical connectivity in larger brains. Such a theorized decrease in total connectedness is supported by empirical results of a lower fraction of cortical neurons that project directly into the white matter in larger brains (Herculano-Houzel et al. 2010). Constraints on the total amount of space available may impact, in particular, long-range connections running between distant areas of the brain. Long-range connections have been argued to need larger axon diameters to maintain fast neuronal communication in larger brains (Ringo et al. 1994; Karbowski 2007), and such “expensive” connectivity might be sparser in bigger brains (Herculano-Houzel et al. 2010; Phillips et al. 2015).

Despite these structural constraints, the macroscale network of brain wiring has been suggested to remain efficient across a range of mammals of widely varying brain size (Assaf et al. 2020). How brain network architecture adapts from smaller to larger brains to maintain efficient processing is still a major unanswered question. We examined the effects of scaling on the organization of macroscale brain connectivity by comparing brains of 14 primate species across three orders of magnitude in size. We hypothesized that allometric scaling of the white matter has the strongest effect on the organization of costly long-range connectivity. We show a shift in the distribution of projection length of corticocortical connections with increasing brain size, resulting in sparser long-range connectivity in larger brains. We illustrate this by investigating the corpus callosum (CC), the main body of long-range fiber bundles connecting areas across the two hemispheres. A constraint on interhemispheric connectivity through the CC has been suggested to promote independent evolution of intrahemispheric connectivity, prompting higher levels of hemispheric lateralization in larger brains as a potential consequence (Ringo et al. 1994; Kaas 2000; Barrett 2012). We further examine the effects of scaling relationships on brain connectivity and network organization, showing a shift from a global toward a more locally efficient network structure with increasing brain size. Our findings together suggest that brain scaling principles extend to patterns of macroscale brain connectivity and support the notion of consequently higher levels of specialization of brain structure in big-brained species like humans.

## Materials and Methods

### MRI Data

**Figure 1:**
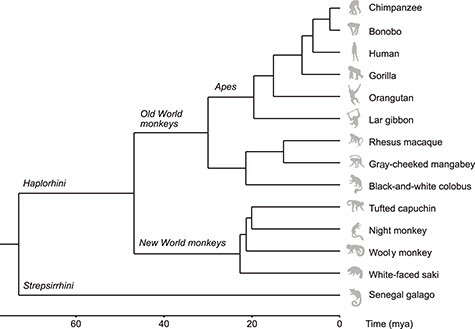
Phylogram of divergence times in million years ago (mya) for the primate species included in this study. Phylogeny was estimated from a consensus tree based on genotyping data of seventeen genes using version 3 of the 10kTrees project (Arnold et al. 2010). Images from phylopic.org.

MRI data of a total of 14 primate species were included (Fig. 1), describing five great ape species (human, chimpanzee, bonobo, gorilla, and orangutan), an ape species (lar gibbon), three Old World monkey species (rhesus macaque, gray-cheeked mangabey, and black-and-white colobus), four New World monkey species (tufted capuchin, night monkey, wooly monkey, and white-faced saki), and a strepsirrhine species (Senegal galago). MRI acquisition protocols, demographics, and data sources are detailed in Table 1. Data were included from an ongoing cohort of MRI scans of brains of the Primate Brain Bank (primatebrainbank.org/data; technical details described in Bryant et al. 2021), the National Chimpanzee Brain Resource (chimpanzeebrain.org), and from previous studies (Rilling and Insel 1999a; Allman et al. 2010; Li et al. 2013; Cabeen et al. 2020). All in vivo data of the National Chimpanzee Brain Resource were acquired prior to the 2015 implementation of US Fish and Wildlife Service and National Institutes of Health regulations governing research with nonhuman primates. Human procedures were approved by Emory University’s Institutional Review Board (IRB00000028), and all participants provided voluntary informed consent. We aimed to maximize the number of species that could be included by collating data from both in vivo and postmortem samples, an approach that has been shown feasible in previous comparative tractography studies (Roumazeilles et al. 2020).

### Structural MRI Processing

Structural MRI scans were processed using FreeSurfer v6.0 (Fischl 2012), including tissue segmentation of cortical gray and white matter, subcortical structures, and reconstruction of cortical surfaces. For nonhuman primate datasets, the FreeSurfer pipeline was complemented with tools from functional magnetic resonance imaging of the brain (FMRIB) software library (FSL) v6.0.1 (Jenkinson et al. 2012), advanced normalization tools (ANTs) (Avants et al. 2011), and Matrix laboratory (MATLAB) to obtain surface reconstructions. Cortical reconstructions were visually inspected for accuracy and consistency across datasets. A brain mask was used to separate brain tissue from any other structures present in the scans, such as skull (in vivo samples) or fiducial markers (postmortem samples). Bias field correction was applied to the images using ANTs. Voxel intensities of T2^*^-weighted images (see Table 1 for datasets) were inverted to create a contrast with low intensity for gray matter voxels and high intensity for white matter voxels. Tissue segmentation was performed using FreeSurfer (Supplementary Fig. S1). Segmentations of white matter, cerebellum, and subcortical structures were visually checked in all species. Manual corrections were made where needed in an iterative process that consisted of visually inspecting and correcting the segmentations and rerunning the subsequent sections of the processing pipeline until high-quality segmentations were obtained (Supplementary Fig. S2). FreeSurfer’s default Talairach registration was complemented by a step-by-step registration process for the smallest brain samples, starting with a registration from the subject at hand to a macaque template brain (Seidlitz et al. 2018), followed by a registration from the macaque template to a chimpanzee template brain (based on the chimpanzee subjects in this study), and from the chimpanzee template to human Talairach space. This registration was reversed to warp an initial tissue probability map from human space to each species’ space in order to aid tissue segmentation. The step-by-step registration process ensured that major brain structures, such as the cerebellum and thalamus, were correctly segmented in all species. Our results were consistent when using alternative, data-driven segmentation strategies using FSL (Jenkinson et al. 2012) (Supplementary Fig. S3). We further verified that the results were not biased by variation in scanning resolution between smaller and larger brains (Supplementary Material).

### Volume and Surface Metrics

Estimates of cerebral volume (i.e., total brain volume except cerebellum and brainstem), cerebral white matter volume, and cortical gray matter volume were obtained from the FreeSurfer segmentation and statistics files of the examined brains. The same files were used to compute total cortical surface area (i.e., area of the folded pial surface separating the brain and the cerebrospinal fluid). The CC, a major white matter tract and the largest interhemispheric bundle in primates (Aboitiz et al. 1992), was clearly identifiable in all examined species. Surface area of the midsagittal slice of the CC has been noted to be a good estimate of total interhemispheric connectivity (Elahi et al. 2015), with fibers running in a consistent direction perpendicular to the sagittal plane. Sagittal slices of the CC were obtained from the T1-weighted MRI data, providing a measure of interhemispheric connectivity that is independent from diffusion-weighted MRI data and the connectivity metrics derived from it. The maximum area of the CC segmentation in the sagittal plane was included as a measure of CC cross-sectional area.

### Cortical Parcellation

Each of the cortical reconstructions was divided into a set of distinct areas randomly placed across the pial surface (Arslan et al. 2018) by using a random parcellation scheme to accommodate the lack of a biologically informed atlas that maps the same, homologous regions in all of the examined primate species. This parcellation procedure consisted of placing a fixed number of region centers evenly dispersed across the cortical mantle. Each vertex in the cortical surface reconstruction was then assigned to the closest region center, resulting in a fixed number of regions of approximately equal size (50 regions per hemisphere; analyses using 25 or 100 regions per hemisphere are described in the Supplementary Material). The parcellation was first created for the left hemisphere and was then also projected onto the right hemisphere using left–right surface registration to achieve left–right symmetry (Greve et al. 2013) (Supplementary Fig. S4). We note that the use of a random atlas does not allow for direct comparison of individual regions across species in terms of anatomical location or function but that it does ensure that resulting connectivity maps include the same number of evenly spaced regions across species. Furthermore, the used implementation defined matching brain areas across the two hemispheres within each species, allowing for within-species comparisons of connectivity profiles (Mars et al. 2016) of spatially corresponding regions across the left and right hemispheres.

**Table 1:** Demographics, MRI scan parameters, and sources of the datasets used in this study

In vivo													
Common name	Scientific name	*N* (*N* female)	Age at scan (y ± SD)	Scanner type	Structural sequence	TR/TE (ms)	Voxel size (mm)	Diffusion sequence	TR/TE (ms)	Voxel size (mm)	Gradient strength (s/mm^2^)	Number of directions/number of b0	Source
Human	*Homo sapiens*	58 (58)	48.3 ± 14.4	Siemens Tim Trio 3T	MPRAGE	2600/3.2	1.0	EPI	8500/95	2.0	1000	60/8	Li et al. 2013
Chimpanzee	*Pan troglodytes*	29 (29)	30.1 ± 12.4	Siemens Tim Trio 3T	MPRAGE	2600/3.6	0.8	EPI	5900/86	1.8	1000	60/40	NCBR
Bonobo	*Pan paniscus*	3 (2)	15.8 ± 10.7	Philips NT 1.5T	FFE	19/8.5	0.7	—	—	—	—	—	NCBR
Gorilla	*Gorilla gorilla*	1 (0)	8	Philips NT 1.5T	FFE	19/8.5	0.7	—	—	—	—	—	NCBR
Orangutan	*Pongo pygmaeus*	4 (1)	15.0 ± 4.6	Philips NT 1.5T	FFE	19/8.5	0.7	—	—	—	—	—	NCBR
Gibbon	*Hylobates lar*	4 (2)	18.4 ± 9.1	Philips NT 1.5T	FFE	19/8.5	0.7^a^	—	—	—	—	—	NCBR
Rhesus macaque	*Macaca mulatta*	15 (15)	14.9 ± 6.7	Siemens Tim Trio 3T	MPRAGE	2600/3.37	0.5	EPI	7000/108	1.1	1000	60/1	Li et al. 2013
Postmortem													
Common name	Scientific name	*N* (*N* female)	Age at death (y)	Scanner type	Structural sequence	TR/TE (ms)	Voxel size (mm)	Diffusion sequence	TR/TE (ms)	Voxel size (mm)	Gradient strength (s/mm^2^)	Number of directions/number of b0	Source
Bonobo	*P. paniscus*	1 (0)	adult	Bruker 7T	3D FLASH	50/4	0.19^b^	PGHE	150/35	1.0	1450	40/6	Cabeen et al. 2020
Gorilla	*G. gorilla*	2 (1)	adult	Bruker 7T	3D FLASH	50/4	0.19^c^	PGHE	150/35	1.0	1450	40/6	Cabeen et al. 2020
Orangutan	*P. pygmaeus*	2 (1)	adult	Bruker 7T	3D FLASH	50/4	0.19^c^	PGHE	150/30	0.825	850	30/6	Cabeen et al. 2020
Gray-cheeked mangabey	*Lophocebus albigena*	1 (1)	27	Agilent Directdrive 7T	—	—	—	DW-SEMS	10 000/25	0.6	4000	128/16	Bryant et al. 2021
Black-and-white colobus	*Colobus guereza*	1 (0)	23.7	Agilent Direcdrive 7T	MGE3D	98/7.5	0.3	DW-SEMS	10 000/25	0.6	4000	128/16	Bryant et al. 2021
Tufted capuchin	*Cebus apella*	1 (0)	22	Agilent Direcdrive 7T	MGE3D	98/7.5	0.3	DW-SEMS	10 000/25	0.6	4000	128/16	Bryant et al. 2021
Night monkey	*Aotus lemurinus*	1 (0)	15.3	Agilent Direcdrive 7T	MGE3D	98/7.5	0.2	DW-SEMS	10 000/25	0.4	4000	128/16	Bryant et al. 2021
Wooly monkey	*Lagothrix lagothricha*	1 (1)	8	Agilent Direcdrive 7T	MGE3D	98/7.5	0.3	DW-SEMS	10 000/25	0.6	4000	128/16	Bryant et al. 2021
White-faced saki	*Pithecia pithecia*	1 (0)	4	Agilent Direcdrive 7T	MGE3D	98/7.5	0.25	DW-SEMS	10 000/25	0.5	4000	128/16	Bryant et al. 2021
Senegal galago	*Galago senegalensis*	1 (0)	20.9	Agilent Direcdrive 7T	MGE3D	98/7.5	0.15	DW-SEMS	10 000/25	0.3	4000	128/16	Bryant et al. 2021

Note: If subjects within a dataset were acquired with different sets of parameters, the most common set of scan parameters is reported. Voxel sizes are isotropic unless noted by a superscript letter. TR, repetition time; TE, echo time; NCBR, National Chimpanzee Brain Resource.

^a^0.7 × 0.7 × 0.6.

^b^0.19 × 0.22 × 0.29.

^c^0.19 × 0.22 × 0.33.

### Connectome Reconstruction

DWI datasets were corrected for eddy current, motion, and susceptibility artifacts using FSL (Jenkinson et al. 2012), followed by deterministic fiber tracking and connectome reconstruction using CATO (v2.5, de Lange and van den Heuvel (2021), dutchconnectomelab.nl/cato). Voxel-wise diffusion profiles were reconstructed using generalized *q*-sampling imaging (Yeh et al. 2010) with a tensor model used in the absence of a complex fiber configuration (Romme et al. 2017; de Lange and van den Heuvel 2021). Eight streamline seeds were started from each voxel in the white matter mask, with streamlines propagated along the best-matching diffusion direction from voxel to voxel until at least one of the stop criteria was reached (exited the brain mask, made an angle of > 60°, and fractional anisotropy of < 0.1). The set of cortical areas were used as network nodes, with connectivity between two network nodes defined as the number of streamlines (NOS) reaching both respective cortical areas, resulting in a connectivity matrix describing the reconstructed corticocortical white matter pathways with the NOS of connections taken as a measure of connection strength (results with fractional anisotropy as connection strength are additionally described in the Supplementary Material). Intrahemispheric connections from the left and right hemispheres were included in the analyses.

### Network Metrics

Connectome organization was examined using network metrics that capture different aspects of the network’s global and local topology. These metrics of network organization were computed for each connectome and compared across species.

#### Density

Network density was computed as the number of observed connections divided by the number of all possible connections between regions.

#### Connection Length

Connection length was computed as the average physical distance traveled by the reconstructed fibers of that connection. For each species, the distribution of connection lengths was split into 10 equally sized bins, ranging from 0 mm to the distance between the most anterior and posterior points of the brain. Each bin therefore represented 10% of the anterior–posterior length of the brain, with the first bin containing all fibers that are within 0–10% of the anterior–posterior length, etc. This binning step was performed to obtain a relative measure of connection length normalized to brain size such that the distribution of connection lengths could be compared across species. Any connections longer than the anterior–posterior distance were included in the longest length bin.

#### Characteristic Path Length

We included binary characteristic path length as a metric of global communication capacity of the network (Bullmore and Sporns 2009). It was computed as the minimum number of steps needed to travel from node *i* to node *j*, averaged across all nodes in the network (Watts and Strogatz 1998; Rubinov and Sporns 2010). Lower values of characteristic path length indicate, an on average, lower number of steps needed to traverse the network, which is indicative of higher global network communication efficiency.

#### Clustering Coefficient

We included binary clustering coefficient as a metric of local network organization (Rubinov and Sporns 2010). It was computed as the number of connections between neighbors of each node *i* divided by the total number of possible connections between those neighbors, averaged across all nodes of the network (Watts and Strogatz 1998; Rubinov and Sporns 2010), with higher values indicating more local segregation of the network.

#### Connectivity Asymmetry

Connectivity patterns of the left versus right hemispheres were compared by means of calculating a level of “connectivity asymmetry.” For each dataset, for each region A in the left hemisphere, we extracted the connection strength of all connections between region A and its connected regions in the left hemisphere, referred to as the connectivity profile of A (Mars et al. 2016). The same was done for the spatial homolog A′ of region A in the right hemisphere, resulting in a connectivity profile of A and a connectivity profile of A′. We then computed the difference between these two connectivity profiles by calculating the absolute difference in connection strength for each of the connections in the two profiles. These per-connection difference scores were then averaged to obtain a single connectivity asymmetry score for region pair A–A′. This asymmetry score captures the degree to which connection strength differs along the connections of region A compared with its homolog region A′, with a value of 0 denoting identical (i.e., symmetrical) connectivity profiles of A and A′ and values > 0 denoting asymmetrical connectivity between the two regions. To ensure scores reflected differences in connection strength, we focused on connections present for both regions A and A′. Connectivity asymmetry scores were averaged across all left–right homolog pairs of regions, resulting in a total connectivity asymmetry score for each dataset.

#### Normalization Procedures

To minimize the potential impact of cross-species differences in network density on the computed network metrics, network density was set equal across species for the calculation of characteristic path length, clustering coefficient, and connectivity asymmetry (van Wijk et al. 2010; van den Heuvel et al. 2017); results obtained without setting density equal are additionally described in the Supplementary Material. Characteristic path length and clustering coefficient were normalized using distributions of degree-preserved randomly rewired reference networks (Maslov and Sneppen 2002; Rubinov and Sporns 2010) (1000 reference networks per connectome). Connectivity asymmetry was normalized for any potential cross-species differences in overall connection strength by resampling the weights to a normal distribution with identical mean and standard deviation (SD) (*M* = 1, SD = 0.2) for both hemispheres in each species (Hagmann et al. 2008; Honey et al. 2009; Ardesch, Scholtens, Li, et al. 2019) (for additional analysis based only on presence and absence of connections, see Supplementary Material). The examined metric of connectivity asymmetry thus reflected relative differences in the distributions of connection weights between the left and the right hemispheres within each species, allowing comparison of the resulting normalized values across species.

### Statistical Analysis

Associations between predictor and outcome variables were tested by means of phylogenetic generalized least squares regression (PGLS) (Pagel 1997; Symonds and Blomberg 2014), an extension of ordinary least squares regression that accounts for potential nonindependence of the comparative data due to shared evolutionary history. PGLS was performed with the caper package in R (Orme et al. 2018) using a consensus phylogenetic tree obtained from the 10kTrees project version 3 (Arnold et al. 2010). A Brownian motion model of evolution (Felsenstein 1985) was fitted, modeling phenotypes of species that share a recent common ancestor to be more similar than phenotypes of more distantly related species. The strength of the evolutionary signal was measured as the covariance in the residuals and captured by Pagel’s λ, with values varying between zero (no phylogenetic signal in the residuals) and one (expected covariance under a Brownian motion model of evolution). Similar effects were observed using alternative evolutionary models (Ornstein-Uhlenbeck and early-burst models (Hansen 1997; Harmon et al. 2010) and using a reduced major axis approach (Smith 2009) (Supplementary Material). PGLS analyses of volumetric and surface properties were conducted on log-transformed data such that the regression coefficients could be interpreted as scaling exponents. PGLS analyses of network metrics were conducted on *z*-transformed data, yielding standardized regression coefficients.

### Data Sharing and Code Accessibility

MRI data are available from the sources listed in Table 1. Processed volumetric and connectivity data and the code for data analysis and figures are available at github.com/ardesch/brainscaling. MRI data and segmentations from the primate species included from the Primate Brain Bank are available at primatebrainbank.org/data at open.win.ox.ac.uk/DigitalBrainBank and at zenodo.org/record/5044936.

## Results

### Gray and White Matter

**Figure 2:**
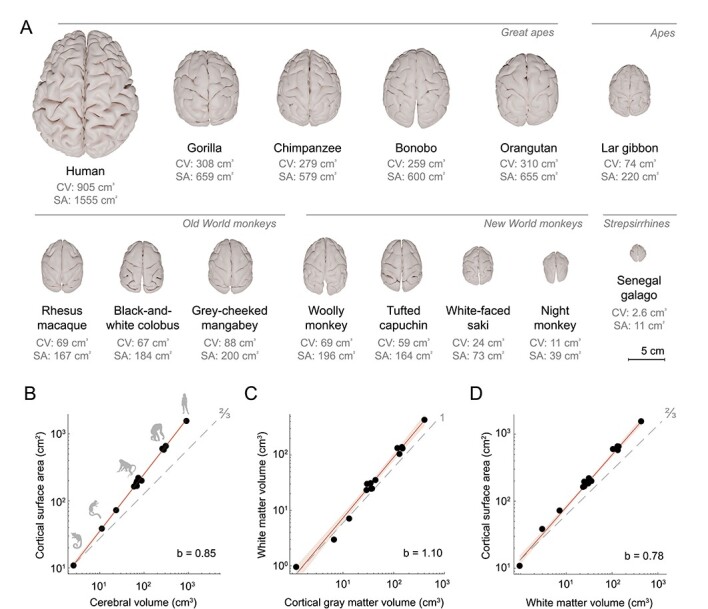
Scaling relationships of the cerebrum. (*A*) Cortical surface reconstructions (to scale). Cerebral volume and cortical surface area were computed from the structural T1 and T2^*^ MRI datasets. (*B*) Scaling between cortical surface area and cerebral volume shows a strong positive allometric relationship. (*C*) White matter volume scales with positive allometry on cortical gray matter volume. (*D*) Cortical surface area scales with positive allometry on white matter volume. In plots (*B*–*D*), the dashed gray line indicates isometric scaling and is annotated with the expected slope for isometric scaling between a surface and a volume (two-thirds) or between two volumes (1). 95% confidence bands are plotted in red to indicate positive allometry. Scaling formula: log(y) = b · log(x) + intercept.

Cerebral volume varied over 350-fold across the 14 species, from 2.6 cm^3^ for the galago to 905 cm^3^ on average for the human subjects (Fig. 2*A*). Cortical surface area varied over 140-fold, between 11 cm^2^ for the galago and 1555 cm^2^ on average for the human subjects. Cortical surface area outpaced cerebral volume with a positive allometric scaling exponent of *b* = 0.85 (95% confidence interval [CI] = 0.82–0.88, adjusted *R*^2^ = 0.99, Pagel’s λ = 0, *P* < 2 × 10^−16^), exceeding an exponent of two-thirds for simple isometric scaling of a 2D surface against a 3D volume (Fig. 2*B*).

White and gray matter volume tended to scale with positive allometry, with a scaling exponent of *b* = 1.10 (95% CI = 0.99–1.21, adjusted *R*^2^ = 0.97, Pagel’s λ = 0.54, *P* = 4.8 × 10^−11^), which corresponds to an increase in the proportion of white matter to cerebral volume from 37% in the galago to 39% in the tufted capuchin, 43% in the gorilla, and 48% in humans (Fig. 2*C*). Cortical surface area outpaced total cerebral white matter volume (*b* = 0.78, 95% CI = 0.73–0.83, Pagel’s λ = 0.60, *P* = 4.2 × 10^−13^) (Fig. 2*D*), indicating that while the proportion of total volume devoted to cerebral white matter is higher in larger brains, it does not keep pace with the rapid cortical expansion with larger brain size.

### Long-Range Connectivity

Overall connectedness decreased with brain size, with network density decreasing from 52% in the Senegal galago to 31–37% in the great apes, including humans (standardized β = −0.76, 95% CI = −1.19 to −0.34, adjusted *R*^2^ = 0.54, Pagel’s λ = 0, *P* = 2.5 × 10^−3^ for the left hemisphere; β = −0.73, 95% CI = −1.18 to −0.28, adjusted *R*^2^ = 0.49, Pagel’s λ = 0, *P* = 4.5 × 10^−3^ for the right hemisphere).

**Figure 3:**
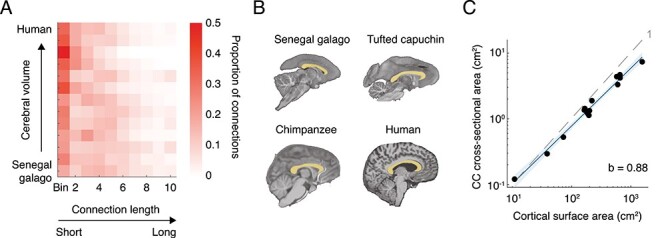
Scaling relationships of long-range connectivity. (*A*) Heatmap showing the proportion of connections that fall in each of 10 connection length bins for the included species. Larger brains (top rows) show low proportions of long connections (light colors in the top-right corner), while smaller brains display a more equal distribution (bottom rows). Length bins were normalized to the anterior–posterior distance of each species’ brain for cross-species comparison, with shorter connections on the left side of the heatmap and longer connections on the right side. The included species are sorted on increasing cerebral volume from the Senegal galago (bottom) to the human brain (top). (*B*) Segmentation of the cross-sectional area of the CC (yellow overlay) in a sagittal slice of the Senegal galago, tufted capuchin, chimpanzee, and human brain (not to scale). (*C*) CC cross-sectional area scales with negative allometry on cortical surface area. The dashed gray line indicates the expected slope of 1 for isometric scaling between two surfaces. 95% confidence band is plotted in blue indicating negative allometry.

We compared the distribution of long versus short connections in each species (normalized to brain size, see Materials and Methods). Larger brains displayed a shift toward lower proportions of long connections and higher proportions of short connections, as compared with smaller brains (Fig. 3*A*). This effect was strongest when contrasting the shortest connections (bins 1–2 in Fig. 3*A*) with longer connections (bins 3–10), with the proportion of shortest connections found to significantly increase with larger brain size (from 36% in the galago and 29% in the night monkey to 41% in chimpanzees and to 60% in humans), while the proportion of longer connections decreased with brain size (from 63% in the galago and 71% in the night monkey to 59% in chimpanzees and to 40% in humans) (β = −0.65, 95% CI = −1.15 to −0.15, adjusted *R*^2^ = 0.36, Pagel’s λ = 0, *P* = 1.7 × 10^−2^).

We examined more specifically the scaling relationship between the cortical surface and the CC (Fig. 3*B*). CC cross-sectional area did not keep pace with cortical surface area, corresponding to a negative allometric relationship, with a scaling exponent of *b* = 0.88 (95% CI = 0.81–0.95, adjusted *R*^2^ = 0.98, Pagel’s λ = 0, *P* = 4.0 × 10^−12^, Fig. 3*C*). This allometric relationship indicates a relatively smaller CC in larger brains, with 1 cm^2^ of CC area available per 90 cm^2^ of cortical surface in the galago but with only 1 cm^2^ of CC area available per 132 cm^2^ of cortical surface in chimpanzees and with only 1 cm^2^ of CC area available per 211 cm^2^ of cortical surface in humans.

### Connectome Organization

**Figure 4:**
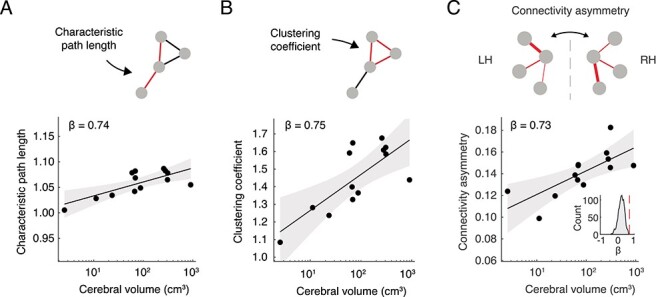
Scaling relationships of network metrics. (*A*) Characteristic path length of the left hemisphere plotted against cerebral volume (normalized to 1000 degree-preserved randomized reference networks). (*B*) Clustering coefficient of the left hemisphere plotted against cerebral volume (normalized to 1000 degree-preserved randomized reference networks). (*C*) Mean connectivity asymmetry of spatially homologous regions in the left versus the right hemispheres, plotted against cerebral volume. Inset: null distribution of the association between connectivity profile asymmetry and cerebral volume after randomly shuffling the connection weights (1000 permutations) (de Lange, Ardesch, et al. 2019). The dashed red line represents the observed regression coefficient as depicted in the main figure. The toy network in the top panel depicts two spatially homologous regions (middle nodes) with asymmetrical patterns of connectivity strength (difference in connection thickness between left and right). In the scatter plots of (*A*–*C*), 95% confidence bands are plotted in gray.

We continued by investigating the effects of brain scaling on global and local network organization. Characteristic path length increased with brain size (β = 0.74, 95% CI = 0.30–1.18, adjusted *R*^2^ = 0.51, Pagel’s λ = 0, *P* = 3.6 × 10^−3^ for the left hemisphere), indicating longer communication paths in larger brains (normalized for total number of connections, Fig. 4*A*). Clustering coefficient also increased with brain size (β = 0.75, 95% CI = 0.32–1.19, adjusted *R*^2^ = 0.52, Pagel’s λ = 0, *P* = 3.0 × 10^−3^ for the left hemisphere), indicating higher levels of local connectivity in larger brains (normalized for total number of connections, Fig. 4*B*). Results were similar for the right hemisphere (characteristic path length: β = 0.66, *P* = 1.4 × 10^−2^; clustering coefficient: β = 0.64, *P* = 1.8 × 10^−2^). Connectomes showed further characteristics of complex brain networks, such as a long-tailed degree distribution, a rich club of highly connected nodes, and an increasing betweenness centrality with larger brain size (Supplementary Fig. 5).

We next examined potential differences in local topology of connectivity of the two hemispheres. We calculated connectivity asymmetry, a normalized measure of connectivity differences between the left and the right hemispheres (Fig. 4*C* top panel, see also Materials and Methods). Connectivity asymmetry was found to be significantly higher in larger brains (β = 0.73, 95% CI = 0.27–1.18, adjusted *R*^2^ = 0.48, Pagel’s λ = 0, *P* = 5.0 × 10^−3^, Fig. 4*C*). Connectivity asymmetry similarly correlated with cortical surface area (β = 0.73, 95% CI = 0.28–1.18, adjusted *R*^2^ = 0.49, Pagel’s λ = 0, *P* = 4.5 × 10^−3^), indicating that connectivity patterns in the left and right hemispheres become increasingly asymmetrical with increasing brain volume and cortical surface area. These effects were controlled for variation in both absolute number and total strength of connections across species, validated across comparison to effects in brain networks with randomly permuted connection strengths (*P* < 2 × 10^−16^, 1000 permutations, Fig. 4*C*, inset), and replicated using alternative measures of connection strength (Supplementary Material).

## Discussion

Our study provides new insights into scaling relationships of major brain components and their consequences for macroscale brain network circuitry in the human and nonhuman primate brains. We validate long-standing evidence of postmortem and comparative MRI studies that white matter volume is outpaced by a disproportionately increased surface area of the cortical mantle, resulting in relatively less space for connectivity to keep cortical areas equally connected in larger brains. Our findings now further characterize this constraint on white matter by showing a reduction in overall connectedness with increasing brain size, particularly of long-range connections. Our comparative study reveals key implications of scaling effects on brain network organization: Connectomes of larger-brained primates—including those of humans—show predictably longer communication paths, higher degrees of clustering, and greater levels of asymmetry between the left and right hemispheres.

The observed relationships between gray matter volume, white matter volume, and cortical surface area corroborate earlier comparative studies (Hofman 1989; Rilling and Insel 1999a, 1999b; Hopkins and Rilling 2000; Mota et al. 2019). Our comparative analysis across a 350-fold range in brain size shows that the proportion of total cerebral volume devoted to white matter connectivity scales exponentially from smaller to larger brains, from around one-third in smaller primates to almost half the volume (48%) in the human brain. The exponent of 1.10 between white matter and gray matter in our data is in line with previous estimates of 1.12 (Rilling and Insel 1999a) and 1.14 (Mota et al. 2019), underlining the robustness of these allometric relationships across different datasets and methodologies.

Despite the higher white matter proportion, our findings show that cortical surface area scales even faster and outpaces white matter volume and the CC, which is closely in line with earlier findings (Rilling and Insel 1999b). Higher proportions of white matter have been proposed to help maintain connectivity in the expanded cortex of larger brains, which requires disproportionately more white matter to remain connected (Deacon 1990; Ringo 1991; Hofman 2012). However, the observed negative allometric scaling exponents of cortical surface area with white matter volume (0.78) and CC cross-sectional area (0.88) indicate that there is less and less space available for white matter connectivity with increasing brain size. Long-range connections in particular appear to be constrained, with larger brains showing relatively more short-range connections than long-range connections when compared with smaller brains. These findings suggest the emergence of a long-range connectivity bottleneck with increasing brain size in primates.

A constraint on long-range white matter connections could prompt connectivity in larger brains to become more space-efficient at the potential cost of longer network communication routes and reduced global information integration. Bigger brains may offset a reduced level of global communication with higher local efficiency as indicated by increasing levels of clustering with brain size. These complementary findings indicate a shift from more global processing in smaller primate brains toward more local processing in larger primate brains, which is in line with theoretical expectations (Ringo 1991; Kaas 2000; Hofman 2012). A clear example of this general trend is the CC. Its relatively smaller size in larger brains leaves less space for long-range communication between the two hemispheres, with higher levels of asymmetry in intrahemispheric connectivity of the left and right hemispheres as a potential consequence. A bottleneck on long-range connectivity has been argued to promote time-sensitive brain functions to be gathered in one hemisphere, leading to increased brain lateralization and specialization (Ringo et al. 1994; Hopkins and Rilling 2000; Kaas 2000; Barrett 2012; Rilling 2014; Hopkins et al. 2015; Phillips et al. 2015). Brain lateralization has been proposed to be an important catalyst for the evolution of specialized and advanced cognitive functions, such as the emergence of complex language and social intelligence in humans (Ringo et al. 1994; Gazzaniga 2000; Roth and Dicke 2005; Rilling et al. 2008; Barrett 2012).

The observed relationships between brain volume, locally specialized connectivity, and global interconnectivity highlight an important trade-off in brain organization (Bullmore and Sporns 2012; Assaf et al. 2020). Overall connectivity and wiring cost have been suggested to be conserved across species, with constraints on interhemispheric connectivity offset by increases in intrahemisperic connectivity (Assaf et al. 2020). Our results complement these findings by showing that, in larger primate brains, the cost-efficiency balance is maintained through enhanced intrahemispheric specialization. Higher levels of lateralization together with lower levels of global connectivity may, however, come at a price of overall reduced network redundancy. Lower network redundancy has been suggested to render the human brain potentially more vulnerable to brain damage, such as stroke (Bartolomeo and Thiebaut de Schotten 2016) and neurodevelopmental disorders (Ribolsi et al. 2009; Bishop 2013; Xie et al. 2018). The CC, one of the largest white matter bundles of the brain and the main bridge between the hemispheres, is regularly reported to be involved in a wide range of both neurological and psychiatric illnesses (de Lange, Scholtens, et al. 2019), including amyotrophic lateral sclerosis (Filippini et al. 2010), schizophrenia (Prendergast et al. 2018), and bipolar disorder (Francis et al. 2016).

The scaling relationships between cortical surface area, gray and white matter volume, and connectivity are likely to be interconnected due to their shared spatial embedding in the brain. Simple scaling rules between cortical surface area and cortical thickness, for example, are argued to underlie the relationships between cortical volume, gray matter, white matter, and the degree of cortical folding across mammalian clades (Zhang and Sejnowski 2000; Mota et al. 2019). Similar interdependencies are observed between cortical surface area, cerebral volume, and various measures of cortical folding, such as gyrification index, folding depth, and folding length (Heuer et al. 2019). These findings suggest that cross-species scaling of neuroanatomical metrics should not be interpreted in isolation but in the context of tightly interrelated development of different brain components. The discussed brain scaling relationships also suggest that human brain organization is not particularly different or “unique” when compared with a general primate trend. Although the human brain is larger than expected for a typical primate of the same body size (Rilling 2014), the contribution of the major tissues to its overall size is predictable (see also Azevedo et al. 2009; Finlay 2019; Miller et al. 2019). We argue that commonly reported and examined aspects of brain network organization and topology may similarly be consequences of brain scaling principles. Our findings of longer communication paths, higher local clustering, and higher connectivity asymmetry with increasing brain size support the notion that the high degree of network specialization observed in humans is a predictable feature of a scaled-up primate brain (Bullmore and Sporns 2012; van den Heuvel et al. 2016; Ardesch, Scholtens, et al. 2019).

A number of methodological factors need to be taken into account when interpreting our findings. The observed scaling relationships do not necessarily generalize to all species (Jyothilakshmi et al. 2020); positive allometry between white and gray matter scaling is a conserved feature in primates but is absent in artiodactyls (hoofed animals including giraffes and deer) (Mota et al. 2019). A second point to consider is that our current analyses are limited to the cerebrum and corticocortical connections. The cerebral cortex has been an important structure of interest in many comparative studies, but the cerebellum has also undergone rapid changes in the evolutionary branch leading up to apes (Miller et al. 2019; Smaers and Vanier 2019). The cerebellum was unfortunately not preserved in some of the postmortem samples we examined and we therefore had to exclude this structure from our analyses. Third, it needs to be mentioned that while diffusion-weighted imaging is one of only a few methods available to obtain information on white matter connections both in vivo and postmortem, and while it tends to show reasonable agreement with more direct and invasive methods such as tract tracing (van den Heuvel et al. 2015; Delettre et al. 2019), diffusion-weighted imaging is well known to suffer from a range of methodological limitations. These limitations result in both false-positive and false-negative fiber reconstructions (Thomas et al. 2014; Maier-Hein et al. 2017), which in turn have their effect on network reconstruction and analysis (de Reus and van den Heuvel 2013; Zalesky et al. 2016). We aimed to minimize the potential impact of false positives by focusing on connections present in both the left and right hemispheres in our weighted network analyses and by averaging the connectivity profile differences across regions. Nevertheless, further investigations using methods at the meso- and microscale will be necessary to specify which types of cortical areas, fiber bundles, and neurons may underlie the connectivity scaling patterns observed on the macroscale.

## Conclusion

Our study shows conserved scaling relationships of major brain components and provides new insights into the expected architecture of macroscale connectivity across primate brain size. Scaling principles of white matter connectivity reveal a constraint on overall brain connectedness, together with a shift towards more local and lateralized processing, in connectomes of larger-brained species. A comparative approach to brain network organization may help identify architectural changes that accompanied brain expansion in recent human evolution.

## Supplementary Material

Rev_SupplementaryMaterial_bhab384

## Notes

We thank prof. John M. Allman (Caltech, USA) for generously sharing the postmortem great ape data with us. *Conflict of Interest*: None declared.
