# Supplementary material for: Scaling Principles of White Matter Connectivity in the Human and Nonhuman Primate Brain
Source: Cereb Cortex. 2021 Nov 24;32(13):2831–42. doi: 10.1093/cercor/bhab384 (PMC9247419; doi:10.1093/cercor/bhab384)
Supplement: Rev_SupplementaryMaterial_bhab384 [file rev_supplementarymaterial_bhab384.zip › Rev_SupplementaryMaterial_bhab384.docx]

**Supplementary Material for**Scaling principles of white matter connectivity in the human and non-human primate brain.

Dirk Jan Ardesch, Lianne H. Scholtens, Siemon C. de Lange, Lea Roumazeilles, Alexandre A. Khrapitchev, Todd M. Preuss, James K. Rilling, Rogier B. Mars, and Martijn P. van den Heuvel

Corresponding author:
Dirk Jan Ardesch, d.j.ardesch@vu.nl

**Supplementary network metrics**

**Degree distribution.** A commonly observed property of many types of complex networks is the presence of a scale-free degree distribution, with many nodes showing relatively few connections (a low degree) and only a few nodes showing many connections (a high degree) (Barabási and Albert 1999). A positively skewed degree distribution has been found in the connectomes of humans and other species (van den Heuvel et al. 2016). We computed the degree distribution across our primate dataset as the number of nodes that have *k* connections for each level of degree *k*, for the left and right hemispheres separately. We then calculated a measure of skewness based on the normalized third moment of the degree distribution (Joanes and Gill 1998), with positive values indicating a positively skewed distribution (a long right tail) and negative values indicating a negatively skewed distribution (a long left tail). All species showed a positively skewed degree distribution (range = 0.013-0.81) except the bonobo, which showed a slightly negatively skewed distribution (-0.079). The skewness of the degree distribution was not significantly correlated with cerebral volume (β = 0.40, 95% CI = -0.20-1.0, adjusted R^2^ = 0.08, Pagel’s λ = 0, P = 0.18 for the left hemisphere, β = -0.29, 95% CI = -0.92-0.34, adjusted R^2^ = -0.001, Pagel’s λ = 0, P = 0.34 for the right hemisphere). These findings suggest that a positively skewed degree distribution is a relatively conserved feature of primate brain networks and that it does not scale strongly with brain size.

**Rich-club organization.** We investigated rich-club organization, a tendency of high-degree nodes to be more densely interconnected than lower-degree nodes (Colizza et al. 2006). For each connectome, we computed the rich-club coefficient for each degree *k* as the number of observed connections between nodes of degree *k* or higher divided by the total number of possible connections between these same nodes (van den Heuvel and Sporns 2011). We compared the observed rich-club coefficients to coefficients obtained in a null distribution of 1,000 degree-preserved randomized reference networks for each connectome. Connectomes were considered to have a rich-club organization if the rich-club coefficient significantly exceeded the null distribution for one or more levels of *k*. Network density was set equal as in the main analysis to reduce possible bias of comparing networks of different density, and intrahemispheric connectivity in the left and right hemispheres was investigated separately. Rich-club organization was observed in all examined species (Figure S4; P < 0.05, FDR-corrected across species and *k*-levels), suggesting that rich-club organization is a prominent feature of brain network organization across primates.

**Betweenness centrality.** We computed node betweenness centrality as the proportion of shortest paths that pass through a given node, indicative of a node’s importance in the network (Rubinov and Sporns 2010). For each network, we calculated the betweenness centrality of all nodes and examined the average betweenness centrality across nodes and the skewness of the per-node betweenness centrality distribution (Joanes and Gill 1998). Values were computed for the left and right hemispheres separately and normalized to a distribution of 1,000 degree-preserved randomized reference networks for each connectome as in the main analysis. Average betweenness centrality correlated positively with cerebral volume (β = 0.75, 95% CI = 0.32-1.2, adjusted R^2^ = 0.53, Pagel’s λ = 0, P = 2.9 × 10^-3^ for the left hemisphere; β = 0.64, 95% CI = 0.14-1.1, adjusted R^2^ = 0.36, Pagel’s λ = 0, P = 0.017 for the right hemisphere), and the betweenness centrality distribution showed a higher skewness in the left hemisphere (β = 0.48, 95% CI = -0.092-1.1, adjusted R^2^ = 0.16, Pagel’s λ = 0, P = 0.09; β = 0, 95% CI = -0.66-0.66, adjusted R^2^ = -0.11, Pagel’s λ = 0, P = 0.99 for the right hemisphere). These findings suggest that the shortest communication paths throughout the network increasingly pass through a limited set of nodes with higher brain size, suggesting a more modular, locally efficient network structure in larger brains.

**Binary connectivity profile asymmetry.** Connectivity profile asymmetry in the main text was computed based on weighted connectivity, taking into account the strength of each connection. This analysis was based on connections present in both hemispheres, resulting in their binary connectivity patterns to be identical. As an alternative, we here computed connectivity profile asymmetry based on binary connectivity (describing the presence/absence of connections without information on connection strength). Region-wise overlap was calculated by computing for each homologous region pair the proportion of overlapping connections between the left and right hemispheres relative to the total number of connections of that region in either hemisphere, varying between 0 (no overlap) and 1 (complete overlap). The mean asymmetry in binary connectivity profiles was then obtained by averaging the region-wise overlap for each dataset and subtracting this number from 1. Correlating binary connectivity profile asymmetry with cerebral volume showed a trend for a positive association (standardized β = 0.51, 95% CI = -0.052-1.08, adjusted R^2^ = 0.19, Pagel’s λ = 0, P = 0.074). This correlation coefficient did not exceed correlation coefficients obtained with a null distribution of degree-preserved randomized networks (P = 0.32, 1,000 randomizations per network). However, connectivity asymmetry was found to scale at a higher rate with cerebral volume in the real networks (unstandardized b = 0.0182) compared with the randomized networks (unstandardized b *M* = 0.0062, SD = 0.0031) (P < 2 × 10^-16^), suggesting that the scaling effect, although detectable in the randomized networks, is much stronger in the real networks. The higher standardized regression coefficient (0.73 vs 0.51) and stronger statistical significance (P = 0.005 vs P = 0.07) of the main weighted analysis compared with the binary analysis suggests that the positive association between connectivity asymmetry and brain size is most strongly driven by differences in the strength of connections between the left and right hemispheres, with potentially a more modest contribution from differences in binary connectivity patterns.

**Network metrics without equal density.** In the main analysis, network metrics were computed based on networks with equal density across species to limit the effect of varying network density on the results (van Wijk et al. 2010; van den Heuvel et al. 2017). Network density was found to decrease with increasing brain size (see main text). We here further explored this density effect on the computed network metrics by repeating the main analyses without setting network density equal. Characteristic path length, clustering coefficient, connectivity asymmetry, and rich-club coefficient were re-computed in an identical manner as described in the main analyses, with the only difference that this time no density threshold was applied. Characteristic path length was found to be positively correlated with cerebral volume (left hemisphere: β = 0.67, 95% CI = 0.19-1.2, adjusted R^2^ = 0.40, Pagel’s λ = 0, P = 0.012, right hemisphere: β = 0.59, 95% CI = 0.042-1.1, adjusted R^2^ = 0.27, Pagel’s λ = 0, P = 0.038). Clustering coefficient was also found to be positively correlated with cerebral volume (left hemisphere: β = 0.75, 95% CI = 0.31-1.2, adjusted R^2^ = 0.52, Pagel’s λ = 0, P = 3.4 × 10^-3^, right hemisphere: β = 0.68, 95% CI = 0.20-1.2, adjusted R^2^ = 0.41, Pagel’s λ = 0, P = 0.011). Average betweenness centrality was associated with cerebral volume (left hemisphere: β = 0.69, 95% CI = 0.21-1.2, adjusted R^2^ = 0.43, Pagel’s λ = 0, P = 9.4 × 10^-3^, right hemisphere: β = 0.59, 95% CI = 0.062-1.1, adjusted R^2^ = 0.29, Pagel’s λ = 0, P = 0.033) but the skewness of the distribution was no longer associated with cerebral volume (P > 0.05 for both the left and the right hemisphere). Connectivity asymmetry showed a positive correlation with cerebral volume (β = 0.60, 95% CI = 0.072-1.1, adjusted R^2^ = 0.30, Pagel’s λ = 0, P = 0.031). Networks further showed rich-club organization in the left and right hemispheres for all included species (FDR < 0.05). These findings indicate that the main results are robust for the use of a density threshold and remain present even when network density varies across species.

**Alternative connection weights.** Diffusion-weighted imaging protocols yield multiple metrics for each reconstructed fiber, each highlighting different aspects of the reconstructed connectivity. The main analysis of connectivity asymmetry was based on the number of streamlines (NOS), a metric that has been related to connection strength (e.g., Ardesch et al. 2019; Assaf et al. 2020). Here we repeat these analyses using fractional anisotropy (FA), another frequently used metric that is thought to relate to fiber microstructure with contributions from myelination and axonal structure (Alba-Ferrara and Erausquin 2013). FA-based connectivity profile asymmetry showed a positive association with cerebral volume (β = 0.60, 95% CI = 0.074-1.13, adjusted R^2^ = 0.30, Pagel’s λ = 0, P = 0.03), exceeding effects of the null condition with randomly permuted connection weights (P = 5.5 × 10^-3^, 1,000 permutations). The FA-based asymmetry scores thus extend the effects reported in the main text by showing a similar association with brain size as the asymmetry scores based on NOS-based connection strength.

**Alternative cortical atlases.** Network analyses can be sensitive to the number of nodes (cortical areas) used (van Wijk et al. 2010). We chose an atlas containing 50 areas per hemisphere in the main text as a reasonable number of areas to use across species (similar to other often-used atlases such as the Desikan-Killiany atlas (Desikan et al. 2006) and the Yeo 17 functional network atlas (Yeo et al. 2011). We here repeated the analyses correlating cerebral volume to connectivity profile asymmetry using atlases of 25 and 100 areas per hemisphere, respectively. Using 25 areas per hemisphere showed a trend for a positive correlation between cerebral volume and connectivity profile asymmetry (β = 0.49, 95% CI = -0.087-1.06, adjusted R^2^ = 0.17, Pagel’s λ = 0, P = 0.09) with this positive correlation being significantly higher than the null model of randomly shuffled connection weights (P = 0.01, 1,000 permutations). Using 100 areas per hemisphere did not show a significant positive correlation (β = 0.31, 95% CI = -0.31-0.94, adjusted R^2^ = 0.02, Pagel’s λ = 0, P = 0.30), although this effect was higher than the correlation obtained in the null model (P = 8.6 × 10^-3^, 1,000 permutations). It is possible that this high number of 100 cortical areas per hemisphere goes beyond the number of cortical areas that may biologically be present, especially in the smaller brains, resulting in a connectivity network with nodes that are not sufficiently distinct from each other. Taken together, these analyses using different cortical atlases are in agreement with the main results in terms of the direction of the correlation, but the strength of the association seems to be at least partly dependent on the number of areas included in the atlas.

**Scanning resolution.** To accommodate their smaller size and still maintain a high level of detail, smaller brains are typically acquired with a smaller voxel size than larger brains, illustrated by a positive correlation between cerebral volume and voxel size in our dataset (T1 data: Pearson’s *r* = 0.81, P = 4.46 × 10^-4^; DWI data: *r* = 0.80, P = 9.99 × 10^-4^). We verified whether this resulted in a comparable scanning resolution across species. The total number of voxels acquired in each brain (i.e., the scanning resolution) was similar across species, with no significant relationship between number of acquired voxels and cerebral volume in the structural MRI data (*r* = -0.26, P = 0.36) or the DWI data (*r* = 0.17, P = 0.59). The number of acquired voxels in the DWI data did not correlate with the observed cross-species differences in connectivity asymmetry (*r* = 0.12, P = 0.69).

**Alternative phylogenetic models.** Different types of statistical models exist that can account for phylogenetic signal in comparative data. We explored the fit of our comparative dataset to three commonly used evolutionary models, namely Brownian motion, Ornstein-Uhlenbeck, and early-burst models (e.g., Heuer et al. 2019). The Brownian motion (BM) model assumes that traits of closely related species are more similar than those of distantly related species (Felsenstein 1985). The Ornstein-Uhlenbeck (OU) model is a modification of the Brownian motion model that additionally introduces a parameter α measuring the strength of attraction towards a theoretical optimum (when α = 0, the OU model reduces to the BM model) (Felsenstein 1988; Hansen 1997). The early-burst (EB) model assumes that traits undergo an “early burst” of evolution early in the phylogenetic tree, with this rate slowing down in time (Blomberg et al. 2003; Harmon et al. 2010). We explored the fit of these three models to the structural brain traits in our comparative dataset using the sample-size corrected Akaike Information Criterion (AICc (Akaike 1998)) as implemented in the geiger package in R (Pennell et al. 2014). Model fit was highly similar across the three models, with a slightly better fit (indicated by lower AICc values) for the Brownian motion model of evolution (Table S1).

In the main text, we calculated scaling exponents between the different traits using phylogenetic generalized least squares (PGLS), a modification of generalized least squares that takes into account the covariance structure as estimated by an evolutionary model such as Brownian motion (Pagel 1997; Symonds and Blomberg 2014). However, least squares methods only model error in the dependent variable and assume the independent variable is measured with perfect accuracy (Smith 2009). This assumption leads to an asymmetric property of the modeled relationship: the slope between x and y is not necessarily the same as the slope between y and x. In comparative studies where both x and y are biological traits that are measured with some degree of error, symmetric methods such as reduced major axis (RMA) regression have been used as an alternative approach (Smith 2009; Bruner et al. 2011). We assessed whether our results were stable when using RMA compared to PGLS. Phylogenetic RMA was carried out using the phytools package in R (Revell 2012), incorporating a Brownian motion model of evolution with Pagel’s lambda estimated by maximum likelihood. Results showed very similar scaling exponents between PGLS and RMA approaches, with cortical surface area outpacing cerebral volume (RMA b = 0.84, PGLS b = 0.85), white matter volume outpacing gray matter volume (RMA b = 1.12, PGLS b = 1.10), cortical surface area outpacing white matter volume (RMA b = 0.78, PGLS b = 0.78), and corpus callosum cross-sectional area being outpaced by cortical surface area (RMA b = 0.87, PGLS b = 0.88).

Together, these results suggest a minimal impact of the choice of evolutionary model (BM, OU, EB) or regression approach (PGLS, RMA) and indicate a high degree of robustness of the allometric findings reported in the main text.

**Robustness of tissue segmentation across species**. We used FreeSurfer (Fischl 2012) to segment the gray and white matter and to create a cortical surface reconstruction from structural MRI scans. This pipeline was initially developed for human MRI scans and contains processing steps that rely on anatomical prior information derived from human brains, such as the Talairach registration step and subsequent automatic segmentation of the subcortical structures (Fischl et al. 2002). We assessed whether the FreeSurfer segmentation process introduced any potential bias in the gray and white matter segmentation of the non-human primates used in our study. We compared FreeSurfer-derived total brain, gray matter, and white matter volumes with the same volumes derived by FSL (Jenkinson et al. 2012), another commonly used neuroimaging pipeline. FSL’s FAST segmentation algorithm only uses voxel intensity information and does not include any (human) anatomical priors, and is therefore purely data-driven (Zhang et al. 2001). We found high concordance between the scaling relationships obtained with both softwares. Total white matter volume and total gray matter volume based on the FSL-derived segmentations were found to scale with an exponent of 1.15 (95% CI = 0.98-1.31, P = 2.9 × 10^-9^), which was very similar to the results obtained with the FreeSurfer-derived segmentations (exponent = 1.13, 95% CI = 1.05-1.20, P = 1.8 × 10^-13^) (Figure S5). Furthermore, a direct comparison of the segmentation volumes obtained with the two approaches showed the regression slope of FreeSurfer- vs FSL-derived volume estimates across species to be very close to 1 (perfect concordance) for total brain volume (b = 0.98, 95% CI = 0.96-1.01, adjusted R^2^ = 0.99, P = 2.1 × 10^-17^), gray matter volume (b = 0.96, 95% CI = 0.89-1.02, adjusted R^2^ = 0.99, P = 6.8 × 10^-13^), and white matter volume (b = 0.99, 95% CI = 0.91-1.07, adjusted R^2^ = 0.99, P = 2.7 × 10^-12^). The confidence intervals include 1 for all three comparisons, showing that at least in our brain scaling relationships, no systematic segmentation bias could be detected.

**Supplementary Table S1**: Model fit of Brownian motion (BM), Ornstein-Uhlenbeck (OU), and early-burst (EB) evolutionary models. Sample-size corrected Akaike Information Criterion (AICc) values are listed for each structural brain trait under different evolutionary models. Lower values indicate better fit.

| **Trait** | **BM** | **OU** | **EB** |
| --- | --- | --- | --- |
| Cortical surface area | 39.24 | 42.55 | 42.44 |
| Cerebral volume | 44.31 | 47.62 | 47.58 |
| Cortical gray matter volume | 43.36 | 46.67 | 46.58 |
| Cerebral white matter volume | 47.02 | 50.33 | 50.33 |
| Corpus callosum cross-sectional area | 35.20 | 38.51 | 38.33 |


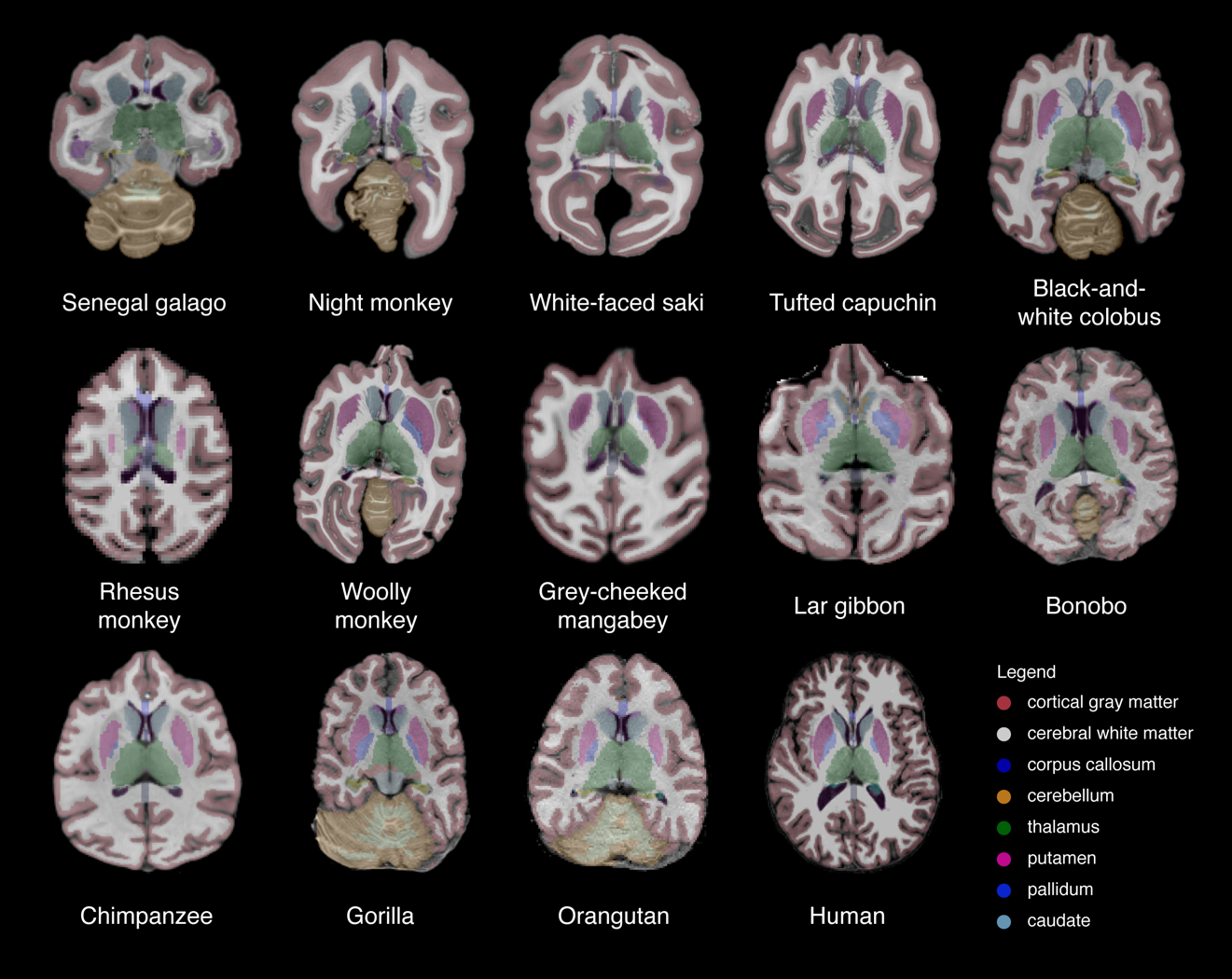


**Supplementary Figure S1:** Segmentation into major brain tissue classes for each species included in the study. The segmentation is overlaid on a bias field-corrected structural volume and depicted in a transverse slice through the thalamus. An exemplary subject was chosen for species with multiple samples/subjects. Brains are visualized at approximately the same size for comparison.

**
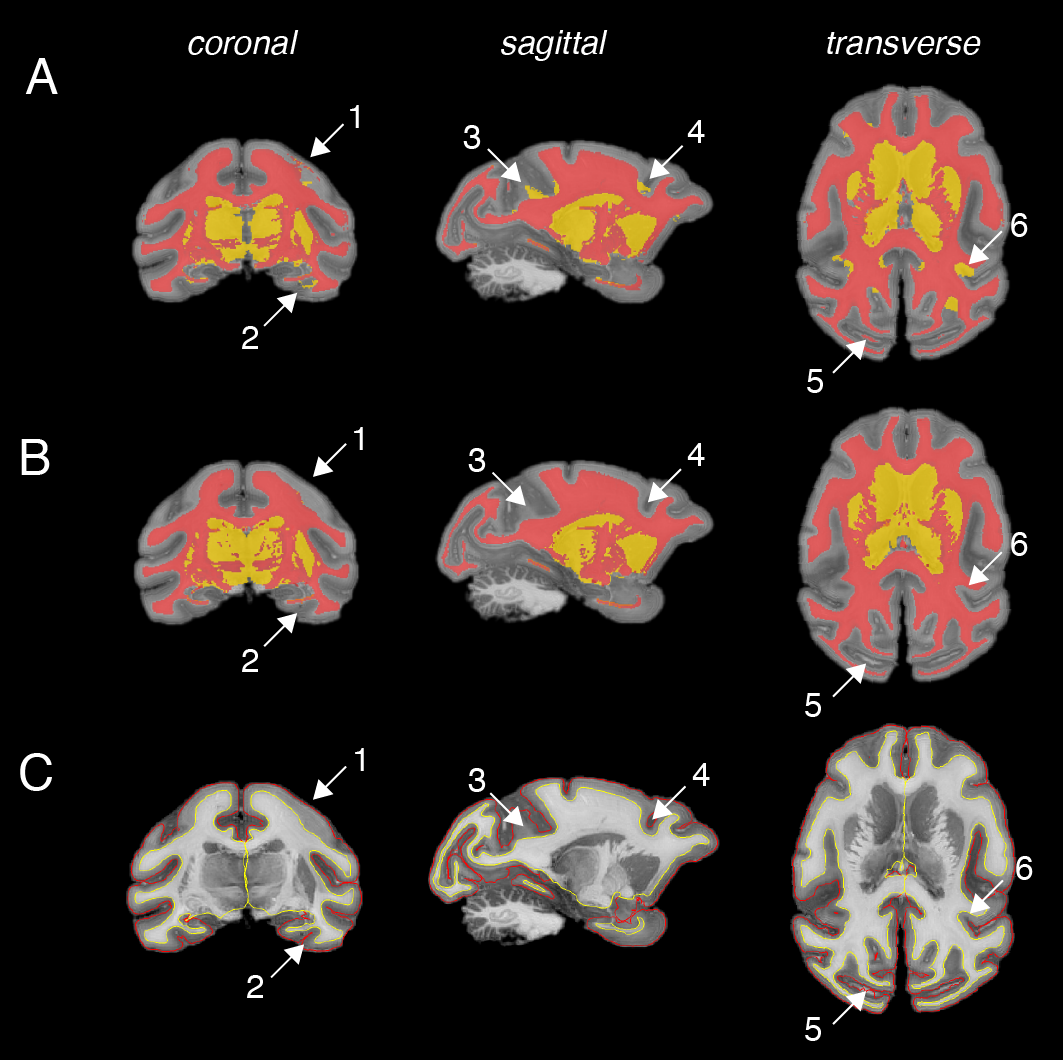
**

**Supplementary Figure S2:** Segmentation example of the tufted capuchin brain. A) Initial white matter segmentation (red and yellow voxels) overlaid on the structural image (grayscale). There is some misplaced white matter in the gray matter (see arrows 1 and 5) and inside sulci (see arrows 2-4 and 6). B) Manually edited segmentation in which these inaccuracies are removed. C) Cortical surface reconstructions of the white matter surface (yellow) and pial surface (red) based on the manually edited segmentations.

**
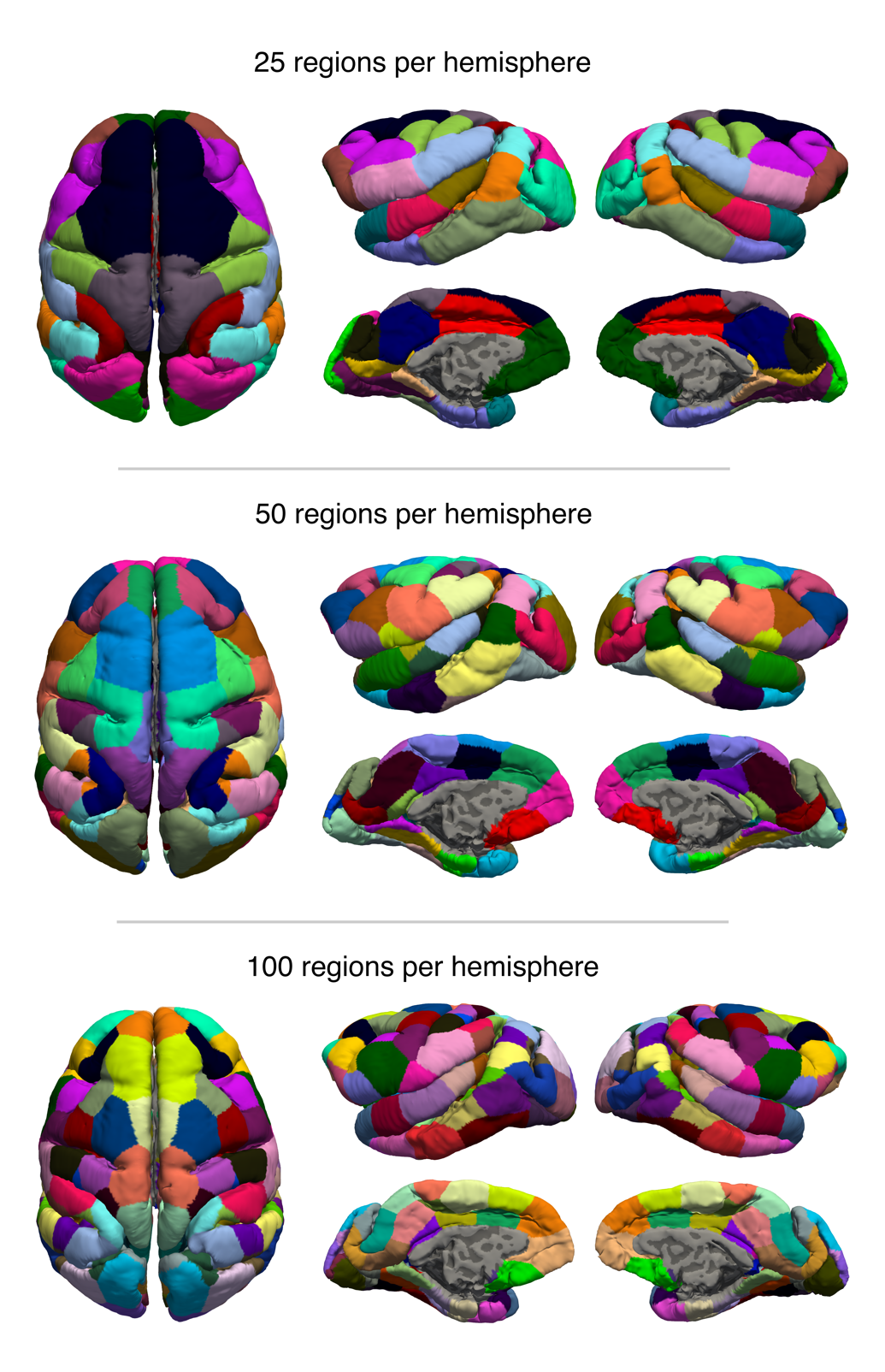
**

**Supplementary Figure S3:** Random left-right symmetrical cortical parcellation. Top-view (left), and lateral/medial views of the left hemisphere (middle) and right hemisphere (right) are shown for the random atlases containing 25, 50, and 100 regions per hemisphere, respectively. The atlas is displayed on the cortical surface of the tufted capuchin.

**
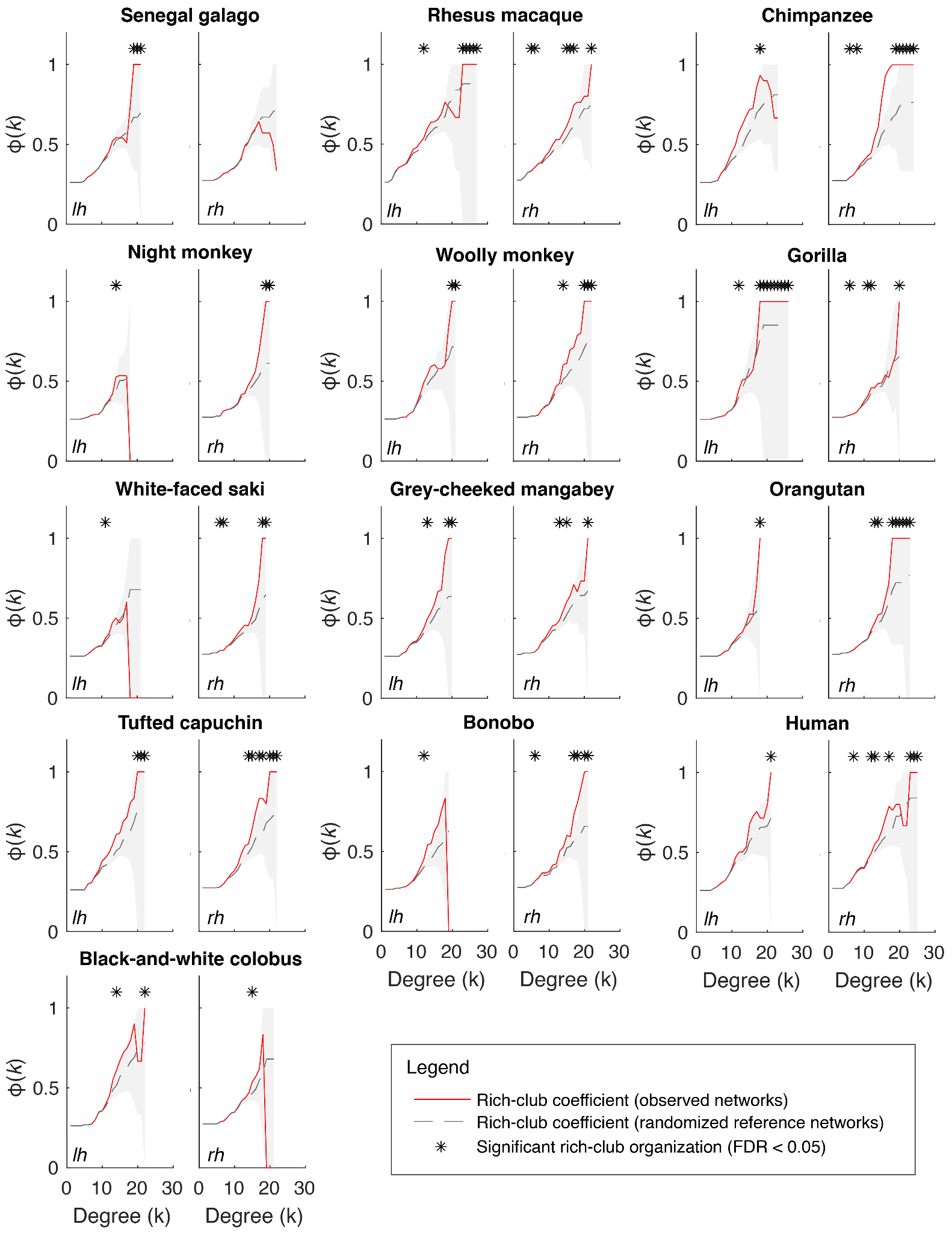
**

**Supplementary Figure S4:** Rich-club organization of primate brain networks. All included species show significant rich-club organization in the left and/or right hemisphere. For species with multiple subjects, data is shown of one randomly chosen subject. The red solid line indicates the observed rich-club coefficient φ for each degree *k*. The grey dotted line depicts the average rich-club coefficient of a null distribution of 1,000 degree-preserved randomized reference networks (grey band denotes the 95% confidence interval of the null distribution at each *k*). Asterisks denote a significantly higher observed rich-club coefficient in the empirical networks than in the null distribution (FDR-corrected across *k*-levels and species). lh: left hemisphere; rh: right hemisphere.

**
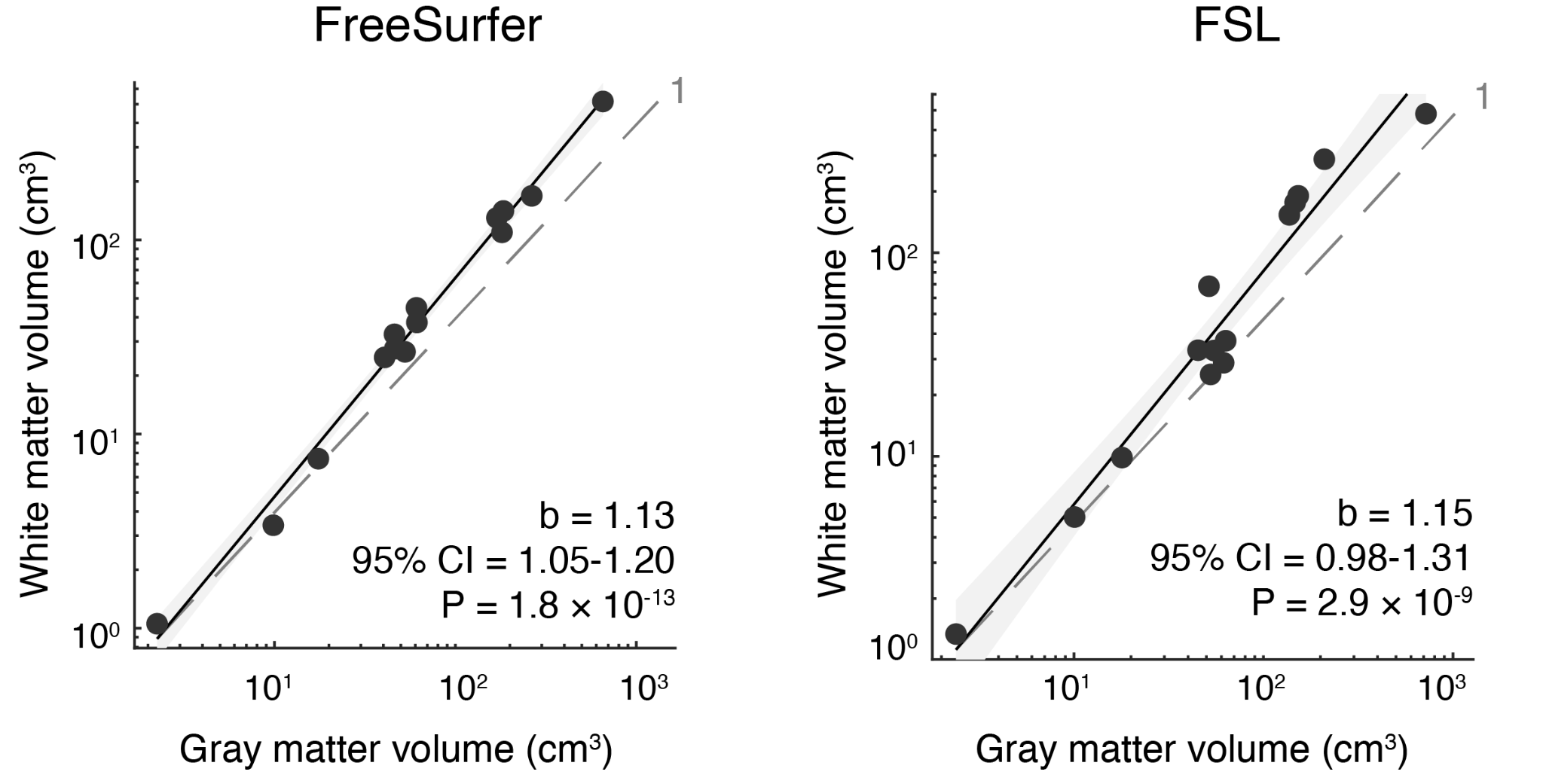
**

**Supplementary Figure S5:** Robustness of scaling relationships to MRI processing method. Scaling relationship between total gray matter volume and total white matter volume is similar when MRI data are segmented using FreeSurfer (left panel) and when segmented using FSL (right panel). Both methods show positive allometric relationships (b > 1) with overlapping 95% confidence intervals. Dashed gray line indicates isometric scaling and is annotated with the expected slope for isometric scaling between two volumes.

**Supplementary References**

Akaike H. 1998. Information Theory and an Extension of the Maximum Likelihood Principle. In: Parzen E, Tanabe K, Kitagawa G, editors. Selected Papers of Hirotugu Akaike. New York, NY: Springer. (Springer Series in Statistics). p. 199–213. https://doi.org/10.1007/978-1-4612-1694-0_15.

Alba-Ferrara LM, Erausquin GA de. 2013. What does anisotropy measure? Insights from increased and decreased anisotropy in selective fiber tracts in schizophrenia. Front Integr Neurosci. 7(3):1–5. doi:10.3389/fnint.2013.00009.

Ardesch DJ, Scholtens LH, Li L, Preuss TM, Rilling JK, Heuvel MP van den. 2019. Evolutionary expansion of connectivity between multimodal association areas in the human brain compared with chimpanzees. Proc Natl Acad Sci. 116(14):7101–7106. doi:10.1073/pnas.1818512116.

Assaf Y, Bouznach A, Zomet O, Marom A, Yovel Y. 2020. Conservation of brain connectivity and wiring across the mammalian class. Nat Neurosci. 23(7):805–808. doi:10.1038/s41593-020-0641-7.

Barabási A-LL, Albert R. 1999. Emergence of Scaling in Random Networks. Science. 286(5439):509–512. doi:10.1126/science.286.5439.509.

Blomberg SP, Garland T, Ives AR. 2003. Testing for Phylogenetic Signal in Comparative Data: Behavioral Traits Are More Labile. Evolution. 57(4):717–745. doi:10.1111/j.0014-3820.2003.tb00285.x.

Bruner E, Cuétara JMDL, Holloway R. 2011. A Bivariate Approach to the Variation of the Parietal Curvature in the Genus Homo. Anat Rec. 294(9):1548–1556. doi:10.1002/ar.21450.

Colizza V, Flammini A, Serrano MA, Vespignani A. 2006. Detecting rich-club ordering in complex networks. Nat Phys. 2(2):110–115. doi:10.1038/nphys209.

Desikan RS, Ségonne F, Fischl B, Quinn BT, Dickerson BC, Blacker D, Buckner RL, Dale AM, Maguire RP, Hyman BT, et al. 2006. An automated labeling system for subdividing the human cerebral cortex on MRI scans into gyral based regions of interest. NeuroImage. 31(3):968–980. doi:10.1016/j.neuroimage.2006.01.021.

Felsenstein J. 1985. Phylogenies and the Comparative Method. Am Nat. 125(1):1–15. doi:10.1086/284325.

Felsenstein J. 1988. Phylogenies and quantitative characters. Annu Rev Ecol Syst. 19(1):445–471. doi:10.1146/annurev.es.19.110188.002305.

Fischl B. 2012. FreeSurfer. NeuroImage. 62(2):774–781. doi:10.1016/j.neuroimage.2012.01.021.

Fischl B, Salat DH, Busa E, Albert M, Dieterich M, Haselgrove C, van der Kouwe A, Killiany R, Kennedy D, Klaveness S, et al. 2002. Whole Brain Segmentation: Automated Labeling of Neuroanatomical Structures in the Human Brain. Neuron. 33(3):341–355. doi:10.1016/S0896-6273(02)00569-X.

Hansen TF. 1997. Stabilizing Selection and the Comparative Analysis of Adaptation. Evolution. 51(5):1341–1351. doi:10.1111/j.1558-5646.1997.tb01457.x.

Harmon LJ, Losos JB, Jonathan Davies T, Gillespie RG, Gittleman JL, Bryan Jennings W, Kozak KH, McPeek MA, Moreno-Roark F, Near TJ, et al. 2010. Early bursts of body size and shape evolution are rare in comparative data. Evol Int J Org Evol. 64(8):2385–2396. doi:10.1111/j.1558-5646.2010.01025.x.

Heuer K, Gulban OF, Bazin P-L, Osoianu A, Valabregue R, Santin M, Herbin M, Toro R. 2019. Evolution of neocortical folding: A phylogenetic comparative analysis of MRI from 34 primate species. Cortex. 118:275–291. doi:10.1016/j.cortex.2019.04.011.

van den Heuvel MP, Bullmore ET, Sporns O. 2016. Comparative Connectomics. Trends Cogn Sci. 20(5):345–361. doi:10.1016/j.tics.2016.03.001.

van den Heuvel MP, Lange SC de, Zalesky A, Seguin C, Yeo BTT, Schmidt R. 2017. Proportional thresholding in resting-state fMRI functional connectivity networks and consequences for patient-control connectome studies: Issues and recommendations. NeuroImage. 152(2):437–449. doi:10.1016/j.neuroimage.2017.02.005.

van den Heuvel MP, Sporns O. 2011. Rich-Club Organization of the Human Connectome. J Neurosci. 31(44):15775–15786. doi:10.1523/JNEUROSCI.3539-11.2011.

Jenkinson M, Beckmann CF, Behrens TEJ, Woolrich MW, Smith SM. 2012. FSL. NeuroImage. 62(2):782–790. doi:10.1016/j.neuroimage.2011.09.015.

Joanes DN, Gill CA. 1998. Comparing measures of sample skewness and kurtosis. J R Stat Soc Ser Stat. 47(1):183–189. doi:10.1111/1467-9884.00122.

Pagel M. 1997. Inferring evolutionary processes from phylogenies. Zool Scr. 26(4):331–348. doi:10.1111/j.1463-6409.1997.tb00423.x.

Pennell MW, Eastman JM, Slater GJ, Brown JW, Uyeda JC, FitzJohn RG, Alfaro ME, Harmon LJ. 2014. geiger v2.0: an expanded suite of methods for fitting macroevolutionary models to phylogenetic trees. Bioinforma Oxf Engl. 30(15):2216–2218. doi:10.1093/bioinformatics/btu181.

Revell LJ. 2012. phytools: an R package for phylogenetic comparative biology (and other things). Methods Ecol Evol. 3(2):217–223. doi:10.1111/j.2041-210X.2011.00169.x.

Rubinov M, Sporns O. 2010. Complex network measures of brain connectivity: Uses and interpretations. NeuroImage. 52(3):1059–1069. doi:10.1016/j.neuroimage.2009.10.003.

Smith RJ. 2009. Use and misuse of the reduced major axis for line-fitting. Am J Phys Anthropol. 140(3):476–486. doi:10.1002/ajpa.21090.

Symonds MRE, Blomberg SP. 2014. A Primer on Phylogenetic Generalised Least Squares. In: Garamszegi LZ, editor. Modern Phylogenetic Comparative Methods and Their Application in Evolutionary Biology: Concepts and Practice. Berlin, Heidelberg: Springer. p. 105–130. [accessed 2021 Mar 11]. https://doi.org/10.1007/978-3-662-43550-2_5.

van Wijk BCM, Stam CJ, Daffertshofer A. 2010. Comparing Brain Networks of Different Size and Connectivity Density Using Graph Theory. Sporns O, editor. PLoS ONE. 5(10):e13701. doi:10.1371/journal.pone.0013701.

Yeo BTT, Krienen FM, Sepulcre J, Sabuncu MR, Lashkari D, Hollinshead M, Roffman JL, Smoller JW, Zöllei L, Polimeni JR, et al. 2011. The organization of the human cerebral cortex estimated by intrinsic functional connectivity. J Neurophysiol. 106(3):1125–1165. doi:10.1152/jn.00338.2011.

Zhang Y, Brady M, Smith S. 2001. Segmentation of brain MR images through a hidden Markov random field model and the expectation-maximization algorithm. IEEE Trans Med Imaging. 20(1):45–57. doi:10.1109/42.906424.
